# Functional Connectivity During Social Reward Processing in Autistic and Neurotypical Adolescents

**DOI:** 10.1002/brb3.71378

**Published:** 2026-04-28

**Authors:** Hua Xie, Dustin Moraczewski, Kathryn A. McNaughton, Katherine R. Warnell, Junaid S. Merchant, Laura A. Kirby, Elizabeth Redcay

**Affiliations:** ^1^ Neuroscience and Cognitive Science Program University of Maryland College Park Maryland USA; ^2^ Department of Psychology University of Maryland College Park Maryland USA; ^3^ Center For Neuroscience Research, Children's National Hospital Washington, D.C. USA; ^4^ The George Washington University School of Medicine Washington, D.C. USA; ^5^ Data Science and Sharing Team, National Institute of Mental Health Bethesda Maryland USA; ^6^ Department of Psychology Texas State University San Marcos Texas USA

**Keywords:** adolescence, autism spectrum disorders, social neuroscience, functional MRI (fMRI)

## Abstract

**Background:**

A core diagnostic feature of autism is atypical social interaction. Reduced social motivation is proposed to underlie these differences. However, empirical neuroimaging studies testing this hypothesis have shown mixed support and have been limited in their ability to understand real‐world social‐interactive processes in autism.

**Methods:**

To address these limitations, we acquired functional MRI data from neurotypical and autistic youth (*n* = 86, 15 females) during a “live” chat that elicits social reward processes. We examined task‐evoked functional connectivity (FC) of regions associated with reward and mentalizing processes within the broader social reward circuitry.

**Results:**

Task‐evoked FC between social reward regions was significantly modulated by social interaction and receipt of social‐interactive reward. Compared to neurotypical peers, autistic youth showed significantly greater task‐evoked connectivity between posterior temporal regions associated with mentalizing and the amygdala, a key node in the reward network. Furthermore, across groups, the connectivity strength between these regions was negatively correlated with self‐reported social motivation and reward during the scanner task.

**Conclusions:**

These findings highlight an important role of FC within the broader social reward circuitry for social‐interactive reward. We suggest that greater context‐dependent FC (i.e., differences between social and non‐social reward) may indicate an increased “neural effort” or hypersensitivity to social feedback that relates to reduced social reward within autistic and neurotypical adolescents. These findings are important because, counter to prevailing theories, they show that greater neural connectivity during social reward during adolescence may confer risk for poor social outcomes.

## Graphical abstract

Heightened task‐evoked functional connectivity was observed in autistic youth between the amygdala and the posterior superior temporal sulcus (pSTS), core regions of the social reward circuitry, on receipt of a social‐interactive reward. The strength of connectivity was negatively correlated with enjoyment of social interaction.

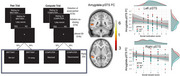

## 1 Introduction

Humans are inherently social creatures, relying on social interactions to survive and thrive, interactions that can be highly rewarding (Krach et al. 2010). Difficulty with social interactions is a core diagnostic criterion for multiple psychiatric conditions, including autism (American Psychiatric Association 2022). One controversial hypothesis is that these social challenges in autism are due to differences in social motivation and social reward processing (Chevallier et al. 2012) that are associated with an atypical neural circuitry (Clements et al. 2018). Thus, determining whether and how the neural substrates of social reward differ in neurotypical and autistic youth is critical to understanding the mechanisms underlying challenges in social interaction.

Numerous neuroimaging studies have examined the neural correlates of social reward processing, which involves the integration of regions within two networks (Ruff and Fehr 2014). The first one is an overlapping cortical‐basal‐ganglia circuitry responsible for processing both monetary and social rewards (Huang et al. 2013). This shared motivational‐reward network includes the striatum, orbitofrontal cortex, anterior cingulate cortex, and bilateral amygdala, supporting the idea of a “common neural currency” theory for reward processing (Montague and Berns 2002; Wake and Izuma 2017). Unlike monetary rewards, social reward processing in neurotypical (NT) participants also engages a broader social‐cognitive network (Ruff and Fehr 2014), including the temporoparietal junction, medial prefrontal cortex, superior temporal sulcus, and temporal pole, which we refer to here as the “mentalizing network.” The mentalizing network is associated with representing others’ mental states and is considered critical for efficient social interactions (Schurz et al. 2014). These two networks also tend to co‐activate during social interaction (Alkire et al. 2018).

Despite theoretical arguments that atypical social reward circuitry may contribute to atypical social interactions in autism, empirical support is mixed (Bierlich et al. 2025; Mcnaughton et al. 2023; Assaf et al. 2013). A meta‐analysis surveying activation studies found that only a little more than half of the studies investigating social reward processing in autism (15 out of 27) showed atypical behavioral and/or physiological responses (Bottini 2018), and additionally, atypical reward processing that is seen is not specific to social reward (Clements et al. 2018; Janouschek et al. 2021).

Several factors may give rise to conflicting findings on the neural substrates of social reward processing in autism. One such factor is the heterogeneity among autistic (AUT) individuals, including the level and display of social motivation (Wing 1997). While autistic individuals may behave and express themselves in idiosyncratic ways, these behaviors do not necessarily indicate differences in motivation or desire for social connection (Jaswal and Akhtar 2018). Some atypical behaviors interpreted as social disinterest could have alternative explanations unrelated to social motivation, such as anxiety and self‐regulation difficulties (Kapp et al. 2011). Many autistic individuals also report high levels of loneliness and often long for friendship (Mazurek 2014)—something that would be inconsistent with a “deficit” in social motivation. Thus, it is important to consider variability in subjective experiences of differences in social motivation and reward.

Another reason for prior mixed findings may be that the experimental paradigms used to study social reward often use non‐interactive or static contexts. For example, a photo of a stranger's smiling face is often used as a social reward, which lacks ecological validity. More recent attempts to increase ecological validity in understanding social processing have typically relied on movie stimuli (Lyons et al. 2020; Turner et al. 2025). While this improves one dimension of ecological validity (i.e., the dynamic and complex nature of social processing), it ignores the critical dimension of social interaction. A large body of work now emphasizes that participation in social interaction (as opposed to mere observation) alters the underlying cognitive and neural processing (Schilbach et al. 2013; Redcay and Schilbach 2019). Therefore, researchers have increasingly advocated for assessing neural processes of social interaction by embedding the brain in a perceived live interactive setting (Redcay and Warnell 2018), which may be critical to eliciting core social processing differences in autism (Rolison et al. 2015). For example, Jasmin and colleagues scanned autistic and typically developing males as they carried out face‐to‐face conversation with an experimenter, and they found autistic males’ cortico‐cortical coupling tends to increase as compared to controls during the conversation (Jasmin et al. 2019; Jasmin et al. 2023).

Relatedly, a potential methodological limitation of past work comes from their analytic approach. While past neuroimaging studies predominately focused on regional activation patterns to study social interaction and social reward, more recent studies have begun to investigate the brain's functional coupling pattern using functional connectivity (FC), which is thought to reflect the brain's interregional communication (van den Heuvel and Hulshoff Pol 2010). Such studies have produced mixed findings on the neural signature of autism, with some reporting weaker connectivity in autistic compared to neurotypical individuals (“hypoconnectivity” in autism), others reporting greater connectivity (“hyperconnectivity”), and some claiming a combination of both dependent on region, task context, and age (Hull et al. 2017; Müller et al. 2011; Roy and Uddin 2021; Yang et al. 2024; Uddin et al. 2013). Studies have also begun to examine FC reconfiguration across brain states in AUT, focusing on the changes of task‐evoked connectivity between task‐evoked conditions and null‐task‐demand conditions to better understand the idiosyncrasy in AUT (Sridhar et al. 2024; Uddin et al. 2015; Barttfeld et al. 2012; You et al. 2013). Moreover, while previous literature highlighted the critical role of the motivational‐reward network and the mentalizing network for social reward processing, the interplay between the two during social‐interactive reward processing in autism has not been systematically investigated.

To address these gaps, we examined how the brain's FC supports reward processing and social interaction, focusing on key brain regions within the social reward circuitry. To maximize ecological validity while maintaining rigorous control over key variables, our experimental paradigm involved an interactive “live” chat, where neurotypical and autistic youth between middle childhood and early adolescence (7–14 years old) shared self‐relevant information and received engaged responses from a peer or computer. This specific age range corresponds with considerable changes in youths’ social competencies and interpersonal relationships (Lam et al. 2014), offering a valuable window to understand underlying neural circuitry (Nelson et al. 2016). We hypothesized that connectivity patterns of the social reward network would be differentially modulated by social‐interactive context and that the AUT group would show different connectivity patterns from the NT group. We further examined whether these differences can be accounted for by the heterogeneity of subjective experiences in social motivation and reward.

## 2 Method

### 2.1 Participants

Sixty‐two autistic youth aged 7–14 years were recruited from fliers, outreach events, the Interactive Autism Network, Simons Foundation Powering Autism Research (SPARK), and a database of local families interested in participating in research. Among them, we excluded those who did not complete the neuroimaging session (*n* = 5); did not believe they were chatting with a real peer partner (*n* = 2, see Section 2.3 Post‐scan interview): were missing more than 1/3 of trials (*n* = 2); and/or had fewer than three usable runs (*n* = 12, i.e., mean framewise displacement (FD) < 0.5 mm). The final autistic sample included 43 autistic youth. We then identified 43 out of a pool of 99 neurotypical youth with a usable MRI scan that matched the autistic sample in mean age, gender, mean full‐scale IQ, and mean in‐scanner motion in terms of framewise displacement (FD). See Table 1 for matched sample characteristics and Supplemental Materials S1 for detailed inclusion criteria. All procedures were approved by the Institutional Review Board of the University of Maryland, and parents and youth provided informed consent and assent.

**TABLE 1:** Matched sample characteristics (*n* = 86). ADOS‐2: Autism Diagnostic Observation Schedule, second edition.

	NT (*n* = 43)	AUT (*n* = 43)	Statistics
Gender (#female / #male)	8/35	7/36	*X* ^2^ (1, *N* = 86) = 0.09, *p* = 0.776
Age (mean ± std, range, years old)	11.60 ± 1.90 (8.10–14.71)	11.86 ± 2.01 (7.58–14.95)	*t*(84) = −0.613, *p* = 0.542
Framewise displacement (mean ± std, mm)	0.167 ± 0.068	0.179 ± 0.090	*t*(84) = −0.706, *p* = 0.482
Number of fMRI runs (mean ± std)	3.88 ± 0.324	3.74 ± 0.493	*W*(43, 43) = 1978, *p* = 0.160
Number of trials (mean ± std)	88.35 ± 9.91	89.26 ± 11.31	*W*(43, 43) = 2171, *p* = 0.418
Full scale IQ (range)	116.6 ± 14.4 (83–146)	113.4±14.9 (86 – 141)	*t*(84) = 1.010, *p* = 0.316
ADOS‐2 total severity (range)	N/A	6.6 ± 1.7 (4–10)	N/A
Gender (#female/#male)	8/35	7/36	*X* ^2^ (1, *N* = 86) = 0.09, *p* = 0.776
Age (years, mean ± std)	11.60 ± 1.90	11.86 ± 2.01	*t* (84) = −0.613, *p* = 0.542
Mean FD (mm)	0.167 ± 0.068	0.179 ± 0.090	*t*(84) = −0.706, *p* = 0.482
Full scale IQ (FSIQ)	116.6 ± 14.4	113.4 ± 14.9	*t*(84) = 1.010, *p* = 0.316
**Race**	NT (*n* = 43)	AUT (*n* = 43)	Total
Asian	0	2	2
Black or African American	8	3	11
White	25	34	59
More than One Race	9	4	13
Missing	1	0	1
**Ethnicity**			
Hispanic/Latino	5	1	6
Not Hispanic/Latino	36	42	78
Missing/Does not wish to disclose	2	0	2
**Household Income**			
$75,000 or more	37	38	75
$65,000–$75,000	1	2	3
$45,000–$65,000	1	2	3
$35,000–$45,000	1	1	2
Missing/Does not wish to disclose	3	0	3

### 2.2 Experimental Protocol

**FIGURE 1:**
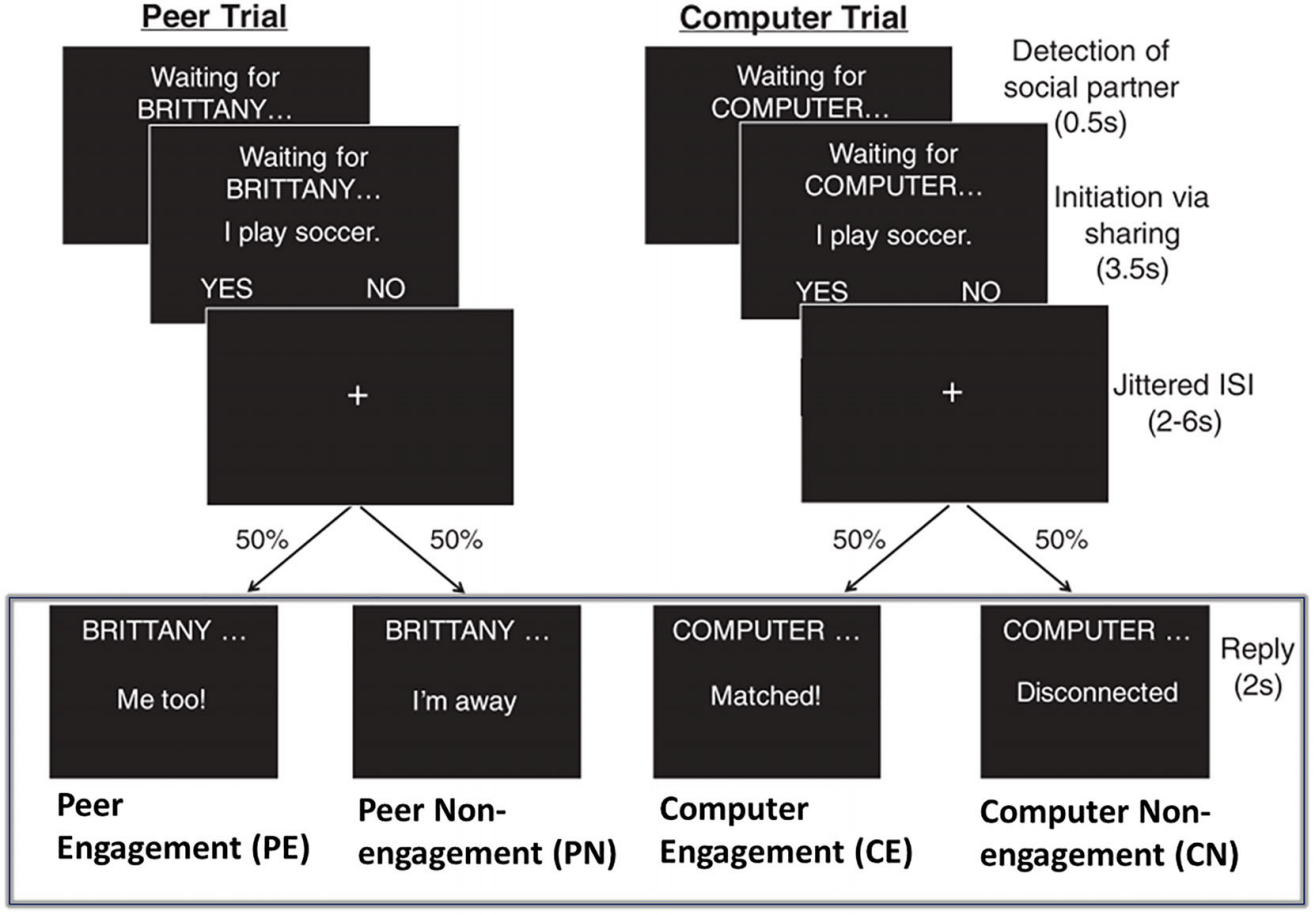
The interactive social interactive task with conditions of interest highlighted by the rectangle. Participants engaged in a text‐based ‘chat’ with a peer or a computer by answering yes or no questions about themselves. The task consisted of two stages, initiation and reply. Here, we focused on the reply stage and investigated three contrasts, that is, social reward contrasts: PE vs. CE and PE vs. PN, and a social context contrast: PE+PN vs. CE+CN (figure adapted from Warnell et al. 2018).

Youth participated in an interactive experiment designed to probe social reward during live social interaction (McNaughton et al. 2023; Warnell et al. 2018). Participants refrained from use of stimulant medications on the day of the scan. As shown in Figure 1, the participant engaged in a text‐based “chat” with an age‐ and gender‐matched peer (who was actually simulated) by answering yes or no questions about themselves (e.g., “I play cookies”) followed by an engaged or disengaged response from the peer. They also completed computer trials, in which they shared information with the computer.

There were four types of reply events: Peer Engagement (PE), Peer Non‐engagement (PN), Computer Engagement (CE), and Computer Non‐engagement (CN), each with six trials per condition per run. The experiment was repeated over four runs, and each run lasted for approximately 6.2 mins. We included participants with three or more usable runs (*n* = 15 with 3 runs and *n* = 71 with 4 runs). The detailed experimental and imaging protocol can be found in Supplemental Materials S2.

### 2.3 Post‐scan Interview

Immediately following the MRI scan, we conducted a verbal interview assessing how much the participants enjoyed interacting with the computer and peers on a 5‐point Likert scale. Two post‐scan enjoyment scores were used in this study, including a social motivation score (the difference between how much they wanted to see the answer from the peer vs. from the computer) and a social reward score (how much they liked chatting with the peer versus computer; see Supplemental Materials S3 for details). We also assessed the participants’ belief in the live illusion by asking them if there was anything else they wanted to tell us. Seventeen participants (*n* = 2 AUT, *n* = 15 NT) who expressed disbelief were excluded from further analysis.

### 2.4 fMRI Data Analysis

#### 2.4.1 Preprocessing

A standardized preprocessing pipeline, fMRIprep v1.4.1, was used to preprocess the imaging data (Esteban et al. 2019). The skull‐stripped BOLD images underwent motion correction, slice timing correction, and susceptibility distortion correction and were lastly resampled to MNI space. Automatic removal of motion artifacts using independent component analysis was performed on the preprocessed fMRI images after removal of non‐steady‐state volumes and spatial smoothing with an isotropic, Gaussian kernel of 6 mm full‐width half‐maximum. Last, the fMRI images were then intensity normalized to have a mean intensity of 1000, and a binary group mask at the threshold of 0.9 probability was applied.

#### 2.4.2 Region of Interests

**FIGURE 2:**
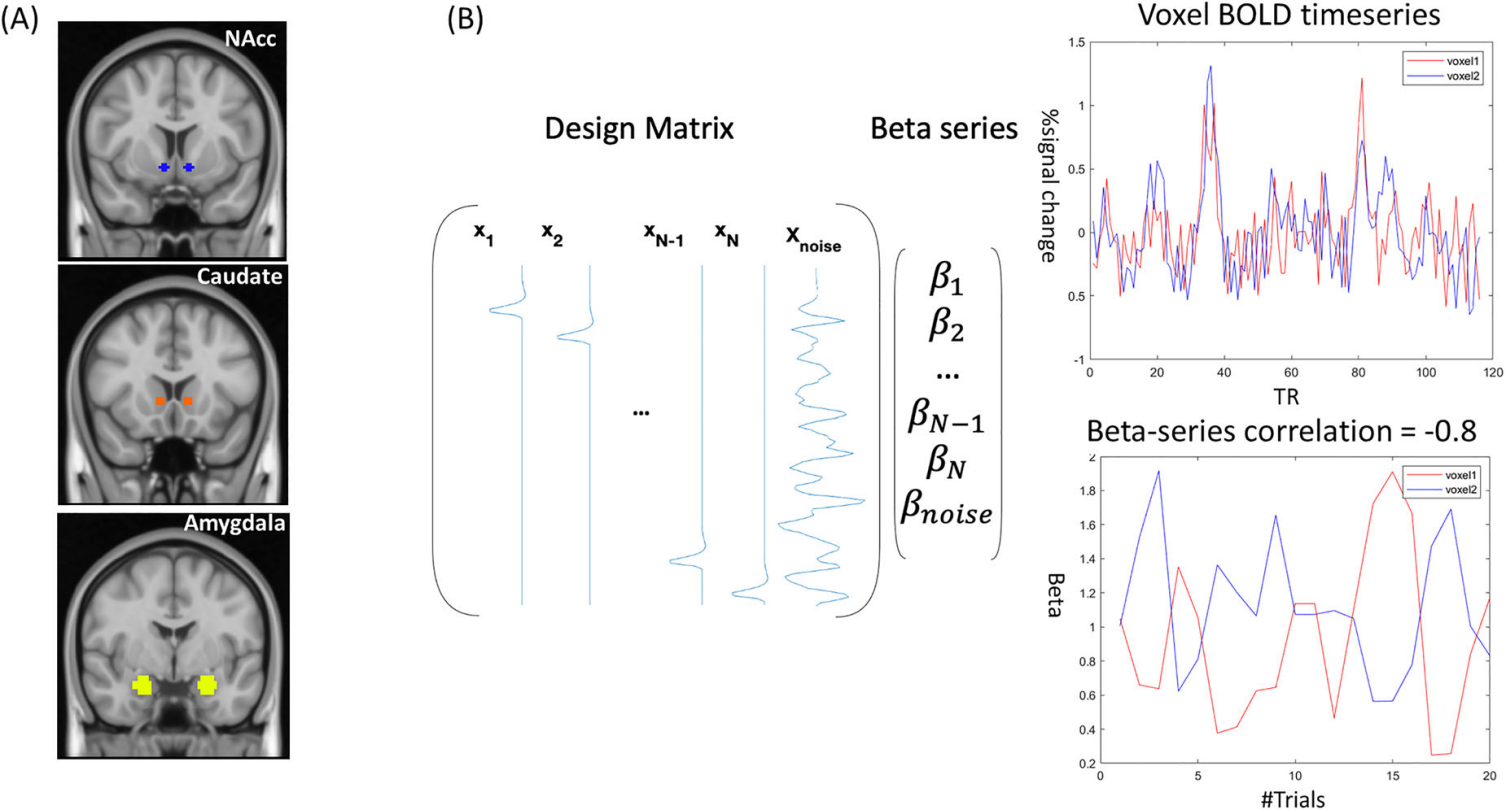
A graphical summary of the context‐modulated FC analysis. (A) Three a priori seed regions are seed regions, that is, nucleus accumbens (NAcc), ventral caudate, and amygdala and (B) Whole‐brain context‐modulated FC analysis. A new set of regressors was created to estimate trial‐specific activation (beta coefficients). The context‐modulated FC was estimated by correlating beta coefficients between the seed and voxels across the whole brain.

Following our previous activation study using the same dataset (Mcnaughton et al. 2023), we chose three a priori seed regions in the motivational‐reward network, that is, the bilateral amygdala, nucleus accumbens (NAcc), and ventral caudate (Figure 2A). The amygdala was anatomically defined in the Harvard‐Oxford subcortical structural probability atlas in FSL, while NAcc and ventral caudate were described in a previous FC‐based study (Di Martino et al. 2011). These regions were chosen given their contribution to social reward processing in autistic (Kohls et al. 2013) and neurotypical youth (Ernst et al. 2005).

#### 2.4.3 Whole‐brain Context‐modulated FC Analysis

We evaluated the whole‐brain context‐modulated FC pattern using the subcortical ROIs as seeds with a beta‐series connectivity approach. An overview of the analysis steps can be found in Figure 2. First, a separate regressor was created for each reply event, which was then convolved with a canonical HRF to model the BOLD response. Second, a trial‐specific activation (beta coefficient) was estimated for each voxel using the Least Squares‐All (LS‐A) approach (Mumford et al. 2012), generating trial‐wise regressors to identify trial‐specific activation. We also censored the frames with FD greater than 1 mm. Third, the beta series were averaged within each ROI, and the averaged timeseries were correlated with beta timeseries of all voxels using Spearman correlation to compute the whole‐brain context‐modulated FC. The correlation coefficients were subsequently Fisher‐z‐transformed.

#### 2.4.4 General Linear Model Analysis

To examine the effects of social context regardless of engagement, we examined the composite social context contrast by comparing the peer (PE + PN) to computer (CE + CN). To examine the effects of social reward specifically, we compared PE (i.e., peer engagement)—the social‐interactive reward when participants receive an engaged, positive response from a peer—versus CE (computer engagement), where participants receive a response from a computer. Additionally, we compared PE versus PN (i.e., peer non‐engagement), which identifies response to social reward controlling for social context. For simplicity, we only refer to the PE versus CE contrast for the remainder of the paper when discussing our social reward contrast because we did not observe significant effects in PE versus PN.

For each seed ROI, we conducted group‐level analyses to identify voxels with significant main effects and group differences in the six whole‐brain FC contrasts using the AFNI function *3dMVM* while controlling for age, gender, and the number of runs. To account for the multiple testing of three seeds and three contrasts, significant clusters were determined with a conservative cluster‐wise false positive rate of 0.0056 (0.05/9 seeds, 124 voxels by *3dClustsim* based on average noise smoothness from the residual data) and a voxel‐wise *p*‐value of 0.001.

## 3 Results

### 3.1 Behavioral Results

A two‐way analysis of variance test was used to examine the main effects of interaction partner (social vs. non‐social) and group (NT vs. AUT) as well as their interaction on self‐reported enjoyment. Similar to our prior activation work using a highly overlapping sample (McNaughton et al. 2023), we found a significant effect of social context such that both groups enjoyed interaction with peers more than the computer (*ps* < 0.001), but there was no significant effect of group nor an interaction effect. We also examined the effects of interaction partner and group on the reaction time to respond to the prompts, and we did not observe any significant differences (*p*s > 0.05).

### 3.2 Context‐modulated FC of Social Reward and Social Interaction

**FIGURE 3:**
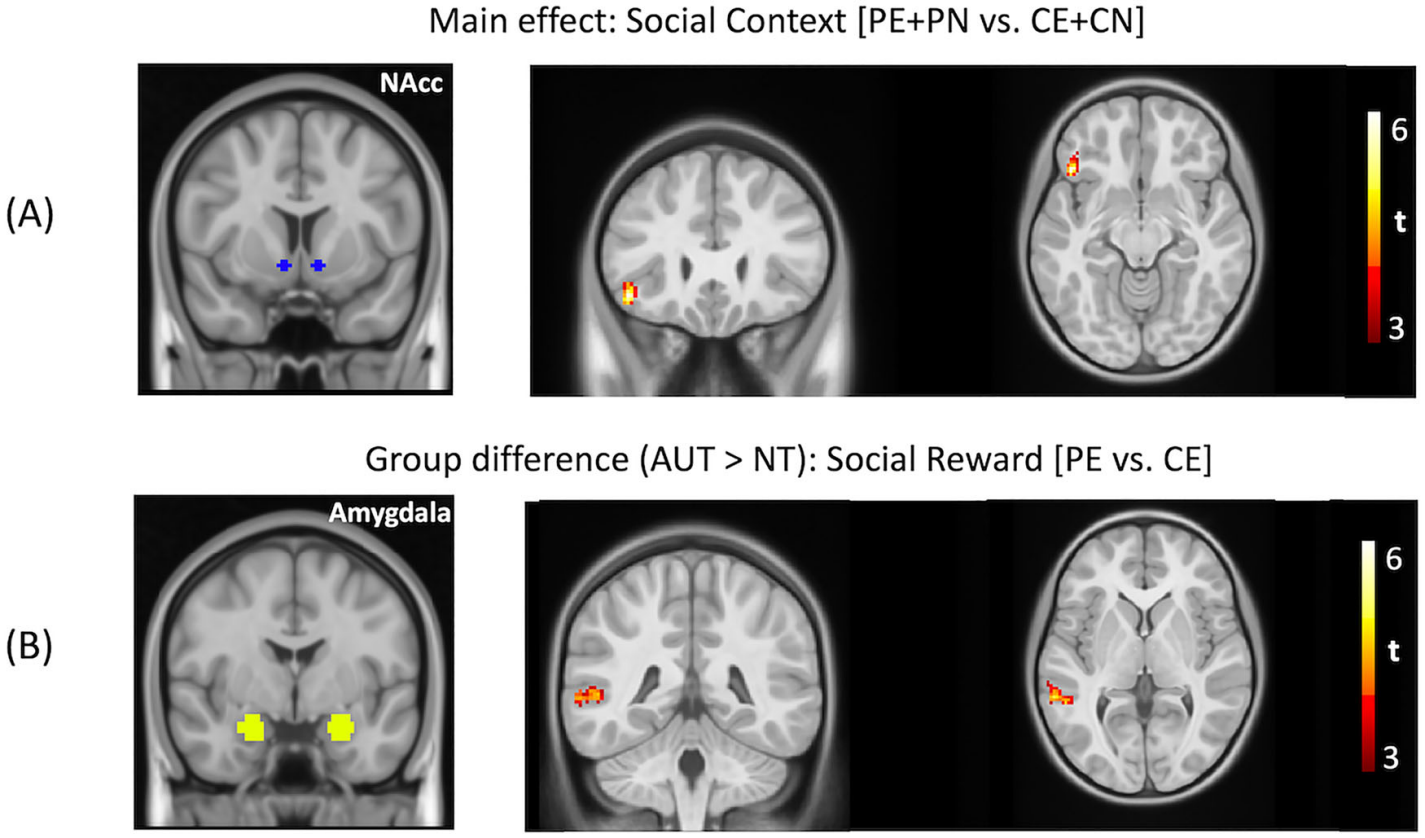
(A) For the main effects, significantly stronger FC was found between the bilateral nucleus accumbens (NAcc) and the left inferior frontal gyrus for the social context contrast. (B) For the group differences, significantly stronger FC was found in the AUT group in the social reward contrast between the bilateral amygdala and bilateral pSTS and right TPJ, respectively (voxel‐wise threshold = 0.001, cluster‐wise threshold = 124 voxels).

We used the trial‐specific beta coefficients of three a priori seed regions. Below we report whole‐brain context‐modulated FC analyses for the social reward (i.e., social engagement vs. non‐social engagement: PE vs. CE) and the social context contrast (i.e., chatting with a peer vs. computer: [PE + PN] vs. [CE + CN]). For the main effect of social context, we observed stronger connectivity between the NAcc and the left inferior frontal gyrus (IFG) during social interaction (Figure 3A). For the social reward contrast [PE > CE], we found significantly stronger FC in the AUT group between the amygdala seed and three regions: bilateral posterior superior temporal sulcus (pSTS) and right temporoparietal junction (TPJ). All analyses were corrected for nine comparisons (3 seeds × 3 contrasts). No other significant effects survived cluster‐wise correction. We also did not observe any significant interaction effect between age and group. A summary of all significant clusters can be found in Supplemental Table S1 in Supporting Materials.

### 3.3 Post‐hoc Analysis on Interaction Enjoyment and Social Anxiety

**FIGURE 4:**
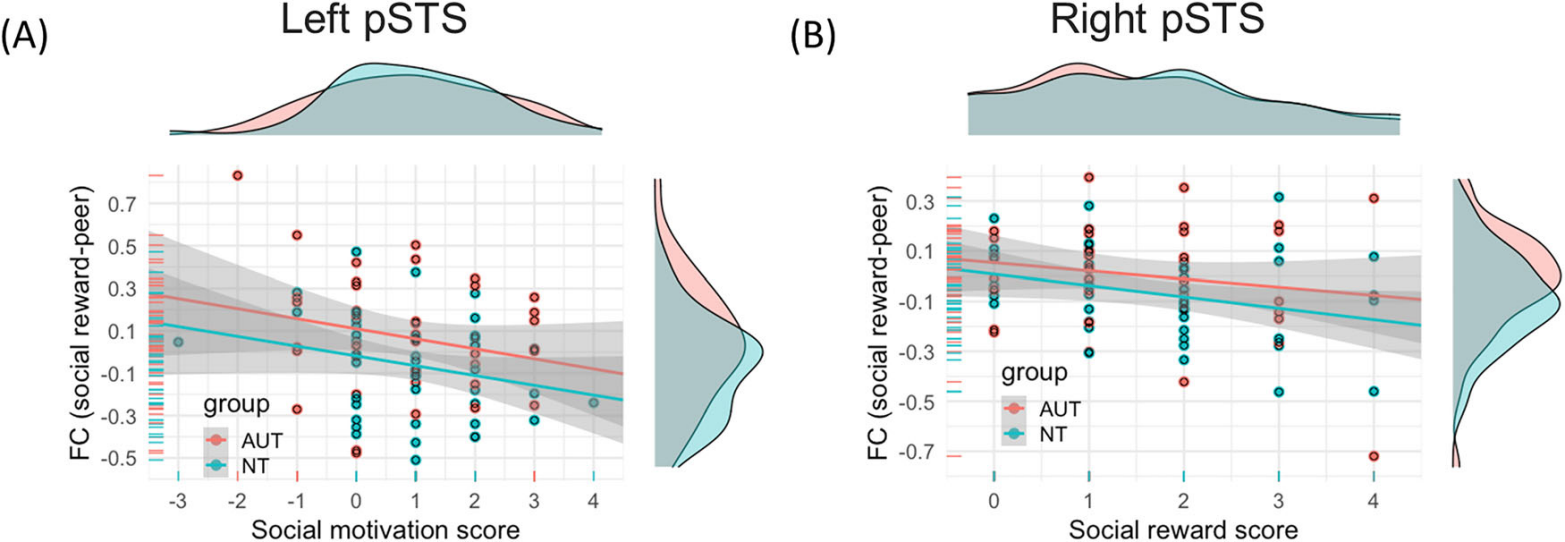
X‐axis: Differences in post‐scan enjoyment scores (peer vs. computer). Y‐axis: FC of social reward contrasts (PE—CE). (A) A significant negative relationship was found between the differences in social motivation score and the FC between the amygdala and left pSTS (*t* = −2.24, *p* = 0.03) and (B) A significant negative relationship was found between the differences in social reward score and the FC between the amygdala and right pSTS (*t* = −2.25, *p* = 0.03).

To explore the brain‐behavioral relationship underlying the significant group differences in context‐modulated FC, we conducted post‐hoc analyses between the FC values of the three significant clusters and post‐scan social reward/motivation scores. We controlled for the diagnosis to ensure that individual differences were not driven by significant group differences. As shown in Figure 4A, after controlling for diagnosis, stronger FC between the amygdala and the left pSTS was related to lower social motivation scores (i.e., differences between how much participants wanted to see the answer from a peer vs. computer) within the combined sample (*t* = −2.24, *p* < 0.05). Similarly, as shown in Figure 4B, FC between the amygdala and the right pSTS was negatively correlated with social reward scores (i.e., the differences between how much participants liked the answer from a peer or computer, *t* = −2.25, *p* < 0.05) after controlling for the diagnosis. All effects remained significant with controlling for the co‐occurrence of attention‐deficit/hyperactivity disorder and the number of comorbidities (see Supplemental Materials S1 for comorbidity information).

To post‐hoc explore if social anxiety drove the elevated connectivity, we correlated FC values between the amygdala and three mentalizing regions with the parent‐reported social phobia scale of the Screen for Child Anxiety Related Emotional Disorders (Birmaher et al., 1997) within a subset of participants (*n* = 56). No significant correlation was found with or without controlling for the diagnosis. Moreover, we reran the analysis after excluding the eight autistic youth with anxiety disorders, and the significant group differences and brain‐behavior relationships with interaction enjoyment remained unchanged.

## 4 Discussion

To understand whether social reward circuitry is modulated by social‐interactive reward and differs between autistic and neurotypical youth, we used a social‐interactive paradigm to investigate task‐induced FC changes during social reward processing in a sample of 86 youth. Middle childhood through early adolescence is a critical developmental period, as difficulties in social interaction during this period predict later mental health difficulties, poorer academic outcomes, and difficulties in later employment (Bornstein et al. 2010; Burt et al. 2008). Given earlier work highlighting the role of FC between or co‐activation of the mentalizing and reward networks during social interaction (Alkire et al. 2018; Assaf et al. 2013; Smith et al. 2014; Xiao et al. 2022), we hypothesized that connectivity profiles of regions associated with reward processing and mentalizing would be modulated by social context and that the AUT group would show different connectivity patterns in these regions. Partially consistent with our hypotheses, we found that social context (i.e., receiving a positive response from a peer compared to a computer) modulated connectivity between reward‐relevant and social‐cognitive regions in the full sample, but these effects did not differ by group. We found significant group differences during social reward processing in connectivity between key regions within the broader social reward circuitry (i.e., encompassing social‐cognitive and reward‐relevant regions of the amygdala and bilateral pSTS and RTPJ). Further, the amygdala‐pSTS connectivity strength was related to individual differences in self‐reported social motivation and reward across groups.

### 4.1 Social Context Relates to Enhanced Mentalizing and Reward Network Connectivity

For the main effect of social context (PE + PN vs. CE + CN, i.e., interacting with a peer vs. computer), consistent with our hypothesis, we found increased connectivity between a core component of the reward system (NAcc) and a part of the mentalizing network (left IFG). Left IFG is an important region associated with mentalizing and empathy (Arioli et al. 2021) and has also been found to encode youth's interest levels in receiving feedback from peers (Guyer et al. 2012). Moreover, activation in IFG and NAcc was differentially modulated during gaze‐based interactions with a perceived human partner compared with a perceived computer (Pfeiffer et al. 2014). Therefore, a potential interpretation of this finding is that strengthened left IFG—NAcc connectivity is related to detecting and updating relevant social signals (particularly from interaction partners), with the NAcc encoding the reward prediction error (Schultz et al. 1997) and the IFG updating expectancies following social feedback (Kohls et al. 2013; Nelson and Guyer 2011).

### 4.2 Stronger Amygdala‐Mentalizing Connectivity During Peer Engagement in AUT

We observed greater connectivity in the AUT group (compared to NT) between the amygdala and bilateral pSTS and nearby RTPJ in the mentalizing network when participants received a positive response from a peer compared to a computer (i.e., social reward contrast [PE vs. CE]). The amygdala is important for social cognition and social motivation (Chevallier et al. 2012; Kohls et al. 2013), and the TPJ and pSTS are key regions for social cognition and social interaction (Redcay et al. 2010). Our findings are consistent with prior neuroimaging studies demonstrating greater connectivity in autism (Jasmin et al. 2019; Jasmin et al. 2023; Uddin et al. 2013; Fishman et al. 2014; Chien et al. 2015; Supekar et al. 2013; Redcay et al. 2013; Dajani and Uddin 2016), especially between subcortical and cortical regions (Cerliani et al. 2015; Ilioska et al. 2022).

Connectivity between pSTS/TPJ and the amygdala has been highlighted in previous neuroimaging studies of reward‐related processing in autism (Dichter 2012). For example, Abrams and colleagues found weaker connectivity between human‐voice‐selective pSTS and the reward circuit, including the amygdala, in autistic children during rest (Abrams et al. 2013), indicating a potential role of amygdala‐pSTS connectivity in reward and human voice processing. In contrast, stronger amygdala‐STS connectivity was found in autism during spontaneous attention to eye gaze in emotional faces during a cognitive control task (Murphy et al. 2012), indicating the role of pSTS‐amygdala connectivity in social information processing.

### 4.3 Stronger Amygdala‐Mentalizing Connectivity Relates to Lower Social Enjoyment

Linking neural data to behavioral data may help us gain a mechanistic understanding of the functional significance of connectivity differences between groups (Picci et al. 2016). Specifically, greater amygdala‐pSTS connectivity during social compared to non‐social reward was negatively correlated with self‐reported social motivation and social reward experienced during the fMRI task. Importantly, social anxiety did not account for greater connectivity to social feedback. Thus, we offer two non‐mutually exclusive possible interpretations for these observations: first, greater connectivity changes between conditions (i.e., FC reconfiguration) index a domain‐general difficulty switching between high‐demanding and low‐demanding conditions; and, second, greater amygdala‐pSTS connectivity to social‐specific feedback reflects hypersensitivity to social reward, which may be related to more negative social experiences.

### 4.4 Greater FC Reconfiguration Indexes Greater Neural Effort?

A first explanation is that the greater FC reconfiguration (i.e., task vs. control FC changes) between regions within the social reward circuitry may signal greater neural effort associated with social interaction (Schultz and Cole 2016). Speaking to this possibility, a previous study found that high‐demand social interaction elicited greater connectivity between social brain regions in autistic compared to neurotypical youth in a live social interaction paradigm (Jasmin et al. 2019). When contrasting the condition with high social demand (conversation) with that of low social demand (repetition), they found stronger task‐evoked connectivity between cortical social processing regions (including STS) in autistic participants, and the increases in connectivity were positively related to the level of social impairment. Greater FC reconfiguration has also been observed in autism during non‐social cognitive tasks. For example, when contrasting a sustained attention task with rest, You and colleagues found that autistic children had increased distal connectivity between frontal, temporal, and parietal regions compared to neurotypical children, and this increased connectivity was associated with inattention problems in everyday life (2013). In addition, Barttfeld and colleagues reported more pronounced connectivity changes in autistic adults than in the neurotypical adults across three cognitive states with varying attention demands (i.e., rest, interoceptive, and exteroceptive attentional states) (2012). Lexical processing induces a similarly greater FC configuration in autistic adolescents as compared with neurotypical peers (Sridhar et al. 2024). Beyond autism, Schultz and Cole found that neurotypical individuals with better task performance had smaller task‐evoked FC reconfiguration when switching from task to rest (2016), suggesting that better‐performing individuals “pre‐configure” their FC at baseline (i.e., rest) to be more efficiently updated for various processing demands. Taken together, greater FC reconfiguration may indicate that social interaction requires greater neural effort and thus is mentally taxing for individuals who find social interaction difficult, regardless of diagnostic status.

### 4.5 Greater Connectivity Indicates Hypersensitivity to Social Reward

A second possible explanation is that greater connectivity of social reward‐relevant regions reflects a neural hypersensitivity to social feedback, which may be related to lower interaction enjoyment. This hypersensitivity to feedback (whether positive or negative) is consistent with growing research demonstrating that adolescence is a time of heightened reward sensitivity to social feedback (Telzer 2016; Orben et al. 2020; Foulkes and Blakemore 2016; Guyer et al. 2012). Further, this heightened sensitivity to reward may be affected by social experiences (Orben et al. 2020; Maza et al. 2023). This finding is consistent with work from neurotypical youth in which greater connectivity between the ventral striatum and anterior temporal lobe during positive social feedback was associated with higher social avoidance goals, presumably due to prior negative social experiences (Davis et al. 2023). The association between social experiences and reward sensitivity is particularly relevant to autistic youth as they experience greater rates of peer rejection and victimization (Rowley et al. 2012), which could affect developing reward systems. Future longitudinal research should include self‐report measures of peer rejection and other social experiences to better understand how prior social experiences may affect hypersensitivity to social reward sensitivity.

### 4.6 Findings Are Specific to Social Context, not Social Reward

Interestingly, we did not find main effects or group differences in the social reward contrast controlling for social context (i.e., PE vs PN). While our activation results with this same paradigm (McNaughton et al. 2023; Warnell et al. 2018) demonstrate ventral striatal activation for the PE versus PN contrast in neurotypical participants, functional connectivity differences in this tightly controlled contrast may be too subtle to detect. Further, because in the PN condition the peer did not respond, it may elicit surprise, which could minimize condition differences. To identify functional connectivity specific to social reward, future studies could contrast functional connectivity during more distinct conditions, such as social versus monetary rewards, for example, using ecologically valid contexts for the social reward.

### 4.7 Limitations and Future Directions

The current study focused on individual differences in processing interactive social reward, including self‐report measures of social motivation and reward. However, the differences in social abilities for autistic youth may also stem from other factors, such as theory of mind, executive function, or sensory processing differences. Moreover, the interactive chat task used in the current study is text‐based, and text‐based communication may alleviate some of the difficulties autistic people experience in face‐to‐face contexts (Benford and Standen 2009). The task also is highly structured, which significantly reduces uncertainty (Boulter et al. 2014). Thus, our highly structured, text‐based interaction may have diminished some potential group differences. Building from our prior work, we focused our seeds on subcortical regions. This identified cortical regions as targets such as pSTS and IFG. However, future studies could use cortical nodes associated with social reward, such as the ventromedial prefrontal cortex, as seeds for a comprehensive understanding of connectivity during social reward processing in autistic and non‐autistic youth. Additionally, given the exploratory nature of our post‐hoc brain‐behavioral analysis, we did not perform multiple comparisons correction, and future work is needed to validate our findings. Although we controlled for age in our analysis, age may add more variance, as previous studies have suggested that autistic youth from different age cohorts may show different connectivity pattern differences (Uddin et al. 2013), which may explain the modest significance level for some of our results. Moreover, while we ensured that our findings were not driven by social anxiety, given the limited sample size and the prevalence of subclinical anxiety in autistic youth (Vasa et al. 2013), future studies with a larger sample of participants with anxiety are needed to fully disentangle the influence of social anxiety on the social reward circuitry. Furthermore, our autistic sample contains few females, which limits the generalizability of these findings, as a recent study demonstrated different patterns of neural sensitivity to social reward in autistic females compared to males (Lawrence et al. 2020).

## 5 Conclusions

In sum, our study demonstrated increased integration between regions associated with reward and mentalizing using a live, reciprocal social interactive paradigm in neurotypical and autistic youth. We found greater task‐based modulation of connectivity within the broader social reward circuitry in autistic youth, which may in turn negatively affect how social‐interactive reward is processed, further highlighting the importance of these networks in studying social interaction in autism.

## Author Contributions

H. X. performed the data analysis, interpreted the data, drafted the manuscript, and critically revised the manuscript. D. M. performed the data analysis and revised the manuscript. K. A. M. interpreted the data and critically revised the manuscript. K. R. W. conceptualized and designed the study and revised the manuscript. J. S. M. revised the manuscript. L. A. K. conducted subject diagnostic and psychometric data collection and revised the manuscript. E. R. conceptualized and designed the study, interpreted the data, critically revised the manuscript, and provided overall supervision of the entire process.

## Funding

Research reported in this publication was supported by the National Institute of Mental Health of the National Institutes of Health under award numbers R01‐MH107441 and R01‐MH125370A awarded to ER and was partially supported by the National Institutes of Child Health and Human Development of the National Institutes of Health under award number P50‐HD105328.

## Conflicts of Interest

The authors declare no conflicts of interest. An earlier preprint version can be found at https://pmc.ncbi.nlm.nih.gov/articles/PMC10274709/.

## Supporting information




**Supplementary Material**: brb371378‐sup‐0001‐SuppMat.docx

## Data Availability

The data that support the findings of this study are openly available through the National Institute of Mental Health Data Archive (NDA) at https://nda.nih.gov/edit_collection.html?id=2394.
