# Supplementary material for: Functional Connectivity During Social Reward Processing in Autistic and Neurotypical Adolescents
Source: Brain Behav. 2026 Apr 28;16(5):e71378. doi: 10.1002/brb3.71378 (PMC13125423; doi:10.1002/brb3.71378)
Supplement: Supplementary file 1 — Supplementary Material: brb371378‐sup‐0001‐SuppMat.docx [file BRB3-16-e71378-s001.docx]

## **Supplemental Materials**

## S1. Inclusion criteria

Youth were excluded if they were born premature (<34 weeks), non-native English speakers, had a history of concussion or head trauma, or had a full-scale IQ < 80 as assessed by the Kaufman Brief Intelligence Test, Second Edition (Kaufman, 2004). Parents of neurotypical youth reported no history of neurological or psychiatric disorders or first-degree relatives with autism or schizophrenia. Autistic youth were not excluded if parents reported a common co-occurring mental health condition, including attention-deficit/hyperactivity disorder (n = 19), obsessive-compulsive disorder (n = 1), anxiety (n = 8), or depression (n = 2). Autistic youth were eligible to participate only if they had a prior clinical diagnosis of autism which was then confirmed by our research team using the Autism Diagnostic Observation Schedule, 2nd edition (ADOS-2, (Lord et al., 2012)) via a licensed clinical psychologist or clinical psychology graduate student who was research-reliable in ADOS administration and coding. Of youth who completed the MRI scan, the following inclusion criteria were used: believed they were chatting with a real peer partner (see section 2.3 Post-scan interview), and adequate task performance (i.e., responding to at least 2/3 of trials) and with three or more usable runs (i.e., mean framewise displacement (FD) < 0.5mm). Twenty-one youth (n = 12 AUT, n = 9 NT) were excluded for excessive motion.

## S2. Experiment and image acquisition

Participants were first informed whether the recipient was a peer (peer trial) or a computer (computer trial), both of which had an equal possibility. Then, the participants initiated an interaction by answering a Yes/No question about their likes and hobbies (e.g., “I like soccer”). After a jittering 2-6 sec (mean 3.5 sec) fixation period, participants received a response in the two-second reply phase. The responses consisted of engagement (e.g., “Me too!”, indicating the peer agreed with the participant, or “Matched!”, indicating that the computer randomly generated the same answer as the child), non-engagement (e.g., “I’m away” or “Disconnected”), and disagreement (e.g., “That’s not what I picked”).

For peer trials, the participants were told that the peer would sometimes be unable to respond because the peer was playing another game. For these disengaged trials, an away message was displayed as the peer response. Moreover, participants believed that the peer always saw their answer, and the peer would either respond if they were able to or not respond if they had been assigned to play another game. For computer trials, participants believed that the computer would randomly pick an answer following participants’ answering the Yes/No question. Moreover, participants were informed that the computer would sometimes lose the connection and be unable to generate an answer, resulting in disengage trials. The order and timing of trials were predetermined, and we used four sets of stimuli to avoid pairing the questions with reply types (e.g., the “I play soccer” trials did not always receive a “Me too” response).

The fMRI data were collected using a single Siemens 3T scanner 32-channel head coil at the Maryland Neuroimaging Center (MAGNETOM Trio Tim System, Siemens Medical Solutions). The scanning protocol consists of four functional runs (T2* weighted gradient echo-planar images; 40 interleaved axial slices; voxel size = 3.0 × 3.0 × 3.0 mm; repetition time = 2200 ms; echo time = 24 ms; flip angle = 78°; pixel matrix = 64 × 64), and one T1-weighted structural scan (176 sagittal slices, voxel size = 1.0 × 1.0 × 1.0 mm; repetition time = 1900 ms; echo time = 2.52 ms; flip angle = 9°; pixel matrix = 256 × 256).

## S3. Post-scan enjoyment questionnaire

A post-scan questionnaire was filled by participants using a 1-5 Likert scale (1 = not at all, to 5 = a lot). The items used in the analysis are listed below.

How much did you want to see his/her answer to your question?

How much did you want to see if the computer matched your answer?

How much did you like chatting with ______?

How much did you like it when you were just answering the computer?

Table S1. Clusters with significant main effect and group differences with MNI coordinates of their center of mass (voxel-wise p-value = 0.001, cluster-wise threshold = 124 voxels).

| Contrast | Seed | Region | x | y | z |
| --- | --- | --- | --- | --- | --- |
| Main effects |  |  |  |  |  |
| Social context | NAcc | L IFG | -45 | 27 | -8 |
| Group differences (AUT > TD) |  |  |  |  |  |
| Social reward | Amygdala | R pSTS | 56 | -61 | 13 |
| Social reward | Amygdala | L pSTS | -59 | -41 | 3 |
| Social reward | Amygdala | R TPJ | -54 | -58 | 20 |
